# Artificial Intelligence, Big Data, and mHealth: The Frontiers of the Prevention of Violence Against Children

**DOI:** 10.3389/frai.2020.543305

**Published:** 2020-10-22

**Authors:** Xanthe Hunt, Mark Tomlinson, Siham Sikander, Sarah Skeen, Marguerite Marlow, Stefani du Toit, Manuel Eisner

**Affiliations:** ^1^Department of Global Health, Institute for Life Course Health Research, Stellenbosch University, Stellenbosch, South Africa; ^2^School of Nursing and Midwifery, Queens University Belfast, Belfast, United Kingdom; ^3^Global Health Department, Health Services Academy, Islamabad, Pakistan; ^4^Institute of Criminology, University of Cambridge, Cambridge, United Kingdom

**Keywords:** violence, child abuse, artificial intelligence, mHealth, big data, machine learning, LMIC

## Abstract

Violence against children is a global public health threat of considerable concern. At least half of all children worldwide experience violence every year; globally, the total number of children between the ages of 2 and 17 years who have experienced violence in any given year is one billion. Based on a review of the literature, we argue that there is substantial potential for AI (and associated machine learning and big data), and mHealth approaches to be utilized to prevent and address violence at a large scale. This *potential* is particularly marked in low- and middle-income countries (LMIC), although whether it could translate into effective solutions at scale remains unclear. We discuss possible entry points for Artificial Intelligence (AI), big data, and mHealth approaches to violence prevention, linking these to the World Health Organization's seven INSPIRE strategies. However, such work should be approached with caution. We highlight clear directions for future work in technology-based and technology-enabled violence prevention. We argue that there is a need for good agent-based models at the level of entire cities where and when violence can occur, where local response systems are. Yet, there is a need to develop common, reliable, and valid population- and individual/family-level data on predictors of violence. These indicators could be integrated into routine health or other information systems and become the basis of Al algorithms for violence prevention and response systems. Further, data on individual help-seeking behavior, risk factors for child maltreatment, and other information which could help us to identify the parameters required to understand what happens to cause, and in response to violence, are needed. To respond to ethical issues engendered by these kinds of interventions, there must be concerted, meaningful efforts to develop participatory and user-led work in the AI space, to ensure that the privacy and profiling concerns outlined above are addressed explicitly going forward. Finally, we make the case that developing AI and other technological infrastructure will require substantial investment, particularly in LMIC.

## Introduction

Violence against children is a global public health threat of considerable concern. At least half of all children experience violence every year; globally, the total number of children between the ages of 2 and 17 years who have experienced violence in any given year is one billion (Hillis et al., 2016). In response to this epidemic, in 2016, 10 international agencies collaborated to produce *INSPIRE: Seven strategies for ending violence against children*, the first-ever global technical package for preventing and responding to violence against children (World Health Organization, 2016). The strategies explain, in detail, how stakeholders can choose and implement interventions that will meet the needs of their context (World Health Organization, 2016).

One of the major causative influences in the development of INSPIRE, and the public health response to violence in general, is the growing scientific evidence that violence has numerous pernicious sequelae, including those which are acute and impact individuals immediately upon being affected (including injury due to physical violence, or trauma due to witnessing a violent incident), as well as those which occur in the longer term. Children exposed to violence in their early years, for instance, experience deficits in socioemotional development, are more likely to experience behavioral problems, and may go on to perpetrate or be victims of violence themselves (Martinez and Richters, 1993; Graham-Bermann and Seng, 2005; Margolin, 2005; Perkins and Graham-Bermann, 2012; Narayan et al., 2017).

Violence has enormous social and economic costs and undermines human capital development. Given these costs, efforts to address violence—to prevent and respond to it—are widespread and diverse (Mercy et al., 1993; World Health Organization, 1996; Butchart et al., 2002; Pronyk et al., 2006; Doll et al., 2007; Shields and Feder, 2016; Ashton, 2020).

Yet, as the problem of violence remains pervasive, there have been calls for innovative solutions (World Health Organization, 1996; Krisch et al., 2015). Among these innovations are those which rely on Artificial Intelligence (AI) (including machine learning), big data, and mHealth approaches to prevent and address violence at a large scale. The potential of these novel technologies to bridge gaps in prevention and response is particularly marked in low- and middle-income countries (LMIC), where infrastructure is often lacking, resources for intervention are scarce, and novel solutions are needed. The potential is so great precisely because the current gaps are so large. However, whether this potential could translate into effective solutions at scale remains unclear.

In this paper, we begin with a mapping review of the literature—from both high-income and low- and middle-income countries—concerning the application of AI, machine learning, big data, and mHealth approaches to the prevention of, and response to, violence. A mapping review (Grant and Booth, 2009) seeks to map out and categorize existing literature, in order to identify gaps in research, and—in the case of this paper—to tabulate findings according to a predetermined framework (INSPIRE). Although mapping reviews are usually focused on a visual synthesis of data based on answering a specific research question, our synthesis is more topic-based (in the manner of a scoping review), given the abundance of fields and diversity of sources from which research on the proposed topic can be drawn.

We then propose how current AI, machine learning, big data, and mHealth innovations may be suited to addressing violence against children in low- and middle-income countries (LMIC), linking these to the World Health Organization's seven INSPIRE strategies.

### Violence as a Public Health Priority

It is increasingly clear that a public health approach is the most effective and sustainable way to address violence (Mercy et al., 1993; Shields and Feder, 2016; Aalsma, 2019; Ashton, 2020), a development which has culminated in the World Health Organization including violence prevention as a public health priority (Resolution WHA49.25 in 1996) (World Health Organization, 1996).

**Figure 1:**
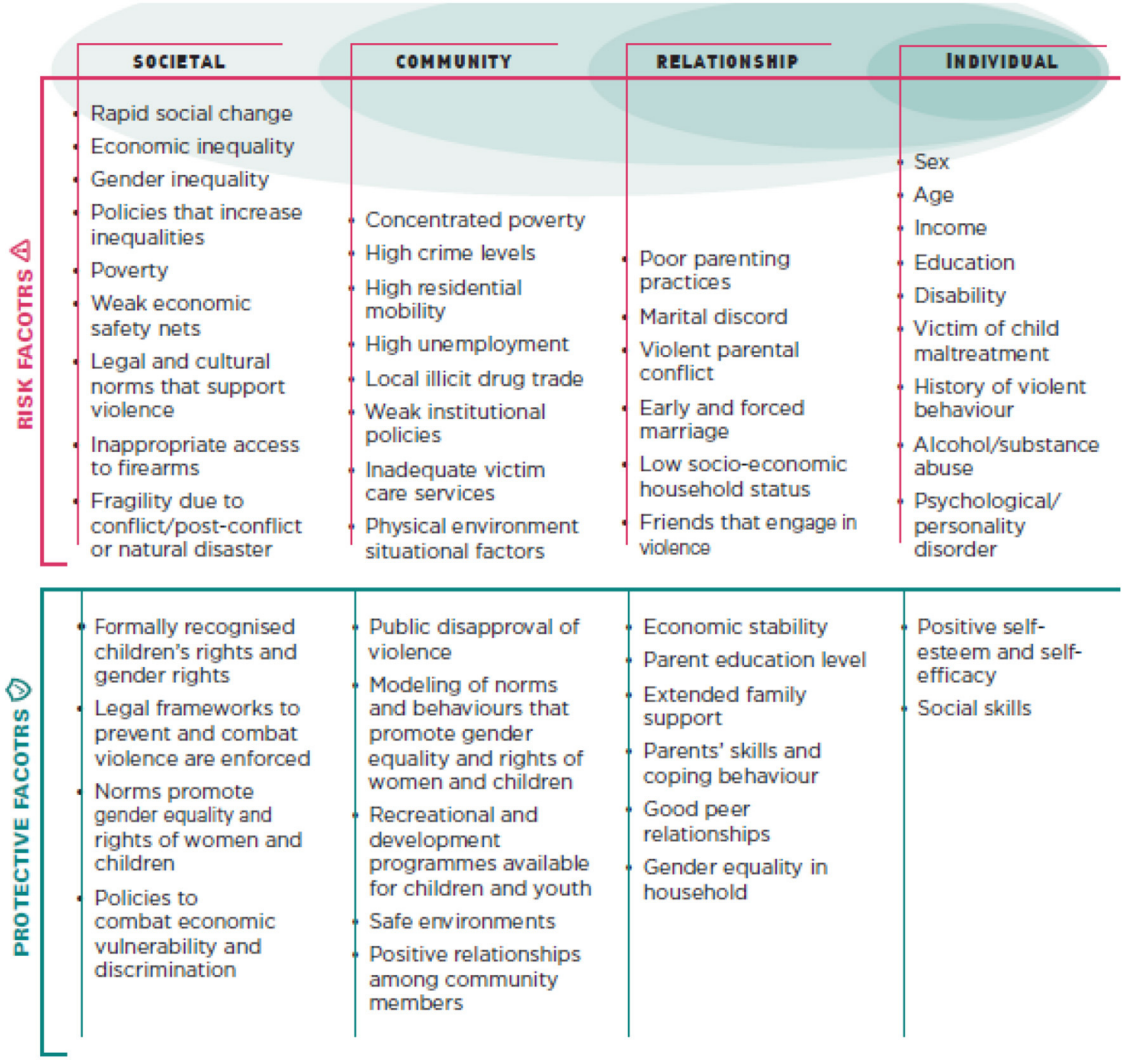
INSPIRE's framework of risk and protective factors (World Health Organization, 2019).

A public health approach is not limited to the public health system, but it is a way of thinking and acting in relation to a given problem. In the case of violence, a public health approach implies that violence needs to be addressed holistically, through policy actions, social change, and attention to the context in which violence is happening (Aalsma, 2019). Figure 1, drawn from the INSPIRE Handbook, shows how risk and protective factors in the environment can be conceptualized, in line with a public health approach. A public health approach accounts for individual risk factors as well as the environmental and social context in which violence occurs, and approaches which are grounded in a public health perspective rely on the identification of contextual factors related to violence (Aalsma, 2019).

Aside from the logic of using a multisectoral, holistic public health-informed approach to address violence, including violence against children, violence is associated with a broad range of issues that are directly relevant for the public health systems. In respect of violence against children, for example, the mental health of mothers and fathers, substance use, malnutrition and neglect, child disabilities, and delayed neurocognitive and psycho-social development are all related (either causally or consequentially) to violence. Violence prevention has many commonalities with global health—violence tends to occur syndemically with other key risk factors for poor health (Meyer et al., 2011), including substance abuse, infectious diseases, and poverty (González-Guarda et al., 2011; Tsuyuki et al., 2017). As such, the prevention of violence, like the promotion of good health and treatment of illnesses and disorders, must attend to a host of comorbidities and commonly co-occurring contextual factors, to be effective.

The final argument which can be made for the relevance of public health to the problem of violence is that, in the case of many sequelae of violence and neglect, actors in the public health system are most likely to see those affected. This includes, for example, health-care visits during pregnancy and health checks in the first years of life, during which health-care workers can counsel against harsh discipline to parents (violence prevention) and monitor children for signs of abuse (violence detection). The health system is also where persons with injuries due to violence will present, and, in many disadvantaged and high-violence areas, the public health system is often more available, more legitimate, and perceived as less corrupt than other systems, especially the criminal justice system. The public health system itself, then, can offer much-needed infrastructure for the delivery of key violence-related services.

In 2002, the World Health Organization released its World Report on Violence and Health, formalizing the marriage of violence and public health and both reflecting and spurring an international consensus to view violence through a public health lens (Krug et al., 2002). On 24 May 2014, the 67th World Health Assembly (WHA) adopted an historic resolution entitled “*Strengthening the role of the health system in addressing violence, in particular against women and girls, and against children*” (World Health Organization, 2014). Through the resolution, which was co-sponsored by 24 governments, the WHA notes that violence persists in every country of the world as a major challenge to public health. The WHO was requested to prepare its first ever global plan of action on strengthening the role of the health system in addressing interpersonal violence, in particular against women and girls, and against children, and finalize its global status report in 2014. The resolution urges member states to ensure that all people affected by violence have timely, effective, and affordable access to health services.

Moreover, in 2016 the World Health Organization together with nine other international organizations active in child protection work launched the “INSPIRE—Seven Strategies for Ending Violence against Children” framework. It is designed to support the work of the Global Partnership to End Violence against Children, and it aims to help governments worldwide to achieve priorities related to violence against children endorsed within the SDGs (World Health Organization, 2016).

A year prior to INSPIRE's release, the World Health Organization and University of Cambridge Global Violence Reduction Conference was held, with the vision of galvanizing political action for the global public health violence prevention field (Krisch et al., 2015). One of the key recommendations stemming from the synthesis of expert opinion from 140 experts was unanimous: there was a need to harness the power of big data in violence reduction (Krisch et al., 2015).

The idea that digital technology be applied to prevent the problem of violence is not new. However, the scale at which such action may be possible is growing; both the complexity of AI, big data, and mHealth technologies, and the variety of social problems to which they can be applied, are expanding. The potential for these innovative solutions to make a difference is particularly marked in low- and middle-income countries (LMIC), where traditional resources for responding to violence are overstretched or non-existent, and the burden of violence is greatest.

AI, ML, Big Data, and mHealth approaches have been taken up with enthusiasm by researchers and interventionists from a number of fields, including global health (Wahl et al., 2018), law enforcement (Mena, 2016), and business (Klumpp, 2018), to name a few. The promise of new technologies to leverage huge amounts of real-time population-level data could be an unprecedented opportunity to prevent crime and violence and to respond when they occur. However, it is currently unclear whether AI and mHealth can support strategies to address violence such as INSPIRE or other frameworks. This especially holds in low- and middle-income countries, where one needs to think carefully about whether AI and mHealth can meet the needs of local actors, and whether there are the resources to implement such strategies.

## Methods

We did not conduct a systematic review to inform this mapping, as the purpose of a mapping review does not require systematic searching (Grant and Booth, 2009). Rather, we purposively searched PsychInfo, PsycARTICLES, Criminal Justice Collection, ProQuest Criminal Justice, and PubMed, and EMBASE for papers published in the last 20 years, on the application of AI, machine learning, Big Data, and/or mHealth (and associated online technologies) to violence prevention or response. Search terms were related to women and children (Population), AI, machine learning, Big Data, and/or mHealth (and associated online technologies) which have been applied to violence prevention or response (Intervention), and any related outcomes (study design and outcome parameters were not set on the search given the descriptive goals of this review). All abstracts were reviewed by the lead author and organized thematically according to (a) type of technology, (b) type of intervention, (c) type of paper (individual study or review), and (d) type of outcomes. Systematic review evidence was prioritized over single empirical papers, and where a study was included in a systematic review already, the primary study was not analyzed in depth.

## Findings

### Current Applications of Artificial Intelligence, Machine Learning, Big Data, and mHealth in Prevention, Early Detection, and Early Response to Violence, Including Violence Against Children

Artificial intelligence (AI) refers to the capacity for learning and “intelligence,” which can be demonstrated by computers and machines. Devices which are described as having AI capabilities are those which can perceive their environment/interpret inputs of various kinds and take actions in relation to these cues in a way which is aligned with their goals/instructions (Nilsson, 2014). Machine learning (ML) can be seen as a subset of AI and refers to computer algorithms that improve automatically through experience (and thus “learn”), improving in their functions as they encounter further inputs (Nilsson, 2014; Marsland, 2015). Big data differs from AI and ML in that it refers to the practice of analyzing large and complex datasets in order to extract meaning from them. While in the past—as the name suggests—the distinguishing feature of big data work was the size of the datasets involved, today big data more commonly denotes specific types of analyses which can be applied to large datasets, including predictive analytics (Boyd and Crawford, 2011). Finally, mobile health (or mHealth) refers to the practice of medicine and public health which is supported by mobile phones (as well as tablets), and the field which is concerned with utilizing smartphones and other communications technologies to deliver health interventions (Kay et al., 2011).

AI and machine learning are computer-based and automated applications of substantial amounts of data on a particular issue. The AI or system utilizes data (of a variety of forms) to apply known instances to make predictions about, and responses to, new instances. In plain language, these technologies use large amounts of data to make predictions and offer options or models based on these predictions. Often, such technologies “learn” over time—adapting how they process information based on the feedback they receive from the environment on their options of models, or as their input information changes. The advantage of using an automated knowledge system is the reduction in human error in decision-making (Reddy et al., 2001), as well as the capacity to utilize huge bodies of data at a pace far exceeding human capacity. Big Data are extremely large data sets that can be analyzed computationally to reveal patterns, trends, and associations, and can be used to inform AI and machine learning.

mHealth can be distinguished from AI in that it utilizes mobile phones, tablets, and other interpersonal communication technologies to address a social problem by communicating or delivering a service to end-users (Harrington, 2018), rather than AI, which works with knowledge and resources, using finite processing capacity, to “learn” and provide solutions to a question [adapted from Wang (2019)]. mHealth interventions, although they may draw on AI, are a different type of product and—in many cases—are simpler in construction and application and do not necessarily rely on large datasets or complex learning algorithms (although they may).

Because of the differences in these two broad classes of innovation, we will be dealing with AI, machine learning, and big data-driven technologies in one section, and mHealth interventions in another.

#### Artificial Intelligence and Big Data

In the fields of public health, public health safety promotion and violence prevention, there are a fair number of examples of AI and big data technologies being used to prevent and control interpersonal violence in high-income countries (HIC). They include strategies to support mental health practitioners, better risk prediction, agent-based modeling, and utilization of online data for violence prevention.

#### Understanding and Addressing Mental Health Issues Associated With Violence

The relationship from violence exposure to mental health conditions is strong and well-established. Exposure to neighborhood-level violence is a predictor of internalizing (for instance, anxious) disorders (Benjet et al., 2019). Exposure to domestic violence is associated with poor mental health outcomes among affected women (Fergusson et al., 2005; Howard et al., 2010), and exposure to violence in childhood is a well-known predictor of a host of negative developmental sequelae (Martinez and Richters, 1993; Graham-Bermann and Seng, 2005; Perkins and Graham-Bermann, 2012) and has long term social, emotional, and behavioral consequences (Margolin, 2005; Narayan et al., 2017). Yet, there is a critical shortfall of psychiatrists and other mental health specialists to provide treatment for people with mental health conditions.

Artificial intelligence is being used by public health practitioners and mental health specialists to assist with the screening, diagnosis, and treatment of mental illness. For instance, Eichstaedt et al. (2018) drew on the history of Facebook statuses posted by 683 patients visiting a large emergency department, analyzing the language used preceding their first documentation of a diagnosis of depression, to identify depressive cases. The model could identify depressed patients to a degree of accuracy which matched screening surveys benchmarked against medical records (Eichstaedt et al., 2018). The University of Pennsylvania's Positive Psychology Center uses machine learning and natural language processing^1^ to analyze social media data and gauge the public's emotional well-being, including levels of depression and trust (Hutson, 2017).

Further, as noted by commentators (Marr, 2019), AI can help support mental health professionals in doing their jobs, including in patient monitoring [as has been done in other sectors, see Davoudi et al. (2018)], and improve accessibility to services. However, its success will depend on the quality of the data sets which inform them, the accuracy of the models, and the capacity of the application to overcome the privacy and other ethical concerns which mark the AI/mental healthcare interface.

#### Space-Based Policing and Information Technologies

There has been a shift toward proactive, predictive, and what is known as preemptive policing in many high-income countries. These efforts rely and build upon surveillance technology (Van Brakel and De Hert, 2011). Rather than responding to a given criminal incident, proactive, predictive or preemptive policing aims to stop crime (and violence) before it happens, by identifying people at risk of perpetration, and monitoring them (National Academies of Sciences, Engineering, and Medicine, 2018). The result of preemptive policing are attempts to better understand the space–time units where certain crimes are most likely to occur and to use resources more effectively to prevent crimes from happening in these locations (National Academies of Sciences, Engineering, and Medicine, 2018). The data-assisted identification of hot spots informs allocation of police [sentinel policing, Nagin (2013)] and strategic placement and monitoring of closed-circuit television cameras (CCTV) (Gandy, 2020).

However, hot-spot policing and CCTV cameras are most applicable to community and/or public space violence. A more family-level application of these processes has been proposed by Daley et al. (2016), who have used “risk terrain modeling” to predict child maltreatment cases. Using analyses of the cumulative effect of environmental factors associated with child maltreatment, the authors created a prediction model to identify future substantiated child maltreatment cases in Fort Worth, Texas (Daley et al., 2016). Their model employed data on aggravated assaults, bars and nightclubs, murders, domestic violence, drug crimes, gang presence, prostitution, poverty, robberies, and runaways prevalences to predict substantiated child maltreatment (Daley et al., 2016). The model performed better than hot-spot mapping, missing only 2% compared to 9% of cases (Daley et al., 2016). This is remarkable because it contradicts the plausible assumption that the spatial concentration of domestic violence or child abuse is driven by mechanisms different from those that generate the concentration of crime in public space.

However, as commentators have been quick to note, the two most important keystones of effective policing are (1) that crimes averted, not arrests made, should be the primary metric for judging police effectiveness and (2) that the publics' views about the police and their approaches and actions matter independently of police effectiveness (Lum and Nagin, 2017). As such, a defining factor in the ongoing effectiveness and sustainability of these approaches will be their ability to prevent crime and be acceptable to the public.

Further, there are immense ethical and moral complexities to the utilization of predictive modeling to identify “potentially criminal” individuals, or even potentially vulnerable groups. There is a great deal of tension between the potential of such initiatives to prevent violence and their potential to contribute to the further marginalization of minorities. It is possible that some of these challenges can be overcome through community involvement and fostering partnerships with minority communities' leadership. However, garnering widespread acceptance from communities concerning how data will be utilized, when those very communities may have histories of marginalization at the hands of authorities, may be extremely difficult.

A final, and less contentious, respect in which space-based predictive models can be utilized in respect of violence concerns the allocation of resources. If hot-spot-type maps are generated for issues other than violence, for instance mental health services or child protection services, health and social inequalities may decrease. Hardt et al. (2013), for instance, used geographic information systems (GIS) mapping software to map health disparities in Alachua County, Florida. Maps were produced for Medicaid births, teen births, low birth weight, domestic violence incidents, child maltreatment reports, unexcused school absences, and juvenile justice referrals (Hardt et al., 2013). The authors used the data to generate “hot spots” density maps of important health and social indicators to highlight where—at the neighborhood level—resources were needed to respond to health inequalities (Hardt et al., 2013).

#### Risk Prediction

Risk prediction in relation to violence against children is not a purely technological activity, nor is it a new one; every child protection officer must make decisions based on an assessment of future risk. Every shelter for abused women makes decisions based on assessments of future risk, and the same holds for foster care, schools, and justice systems.

However, the power of big data to improve the accuracy of risk prediction, combining information on known correlates of violence to produce composite risk indices, is potentially far greater than humans'. Naturally, the sole reliance of prevention workers on risk prediction algorithms as a replacement to human judgment is much debated. As Sreenivasan et al. (2000) describe, risk assessment for violence and sex offender recidivism has been dichotomized into the “clinical approach” vs. the “actuarial method,” where an actuarial only approach uses actuarially derived decisions and predictions alone as a replacement to replace existing clinical practice. This use of a calculated risk score, in the absence of clinical assessment, fails to satisfy many commentators' concerns about public safety, peer accepted standards of practice, liability issues, and concordance with evidence-based medicine practice (Sreenivasan et al., 2000). On the other hand, substantial progress has been made, over the past 20 years, in statistical prediction models for offending and violence (Garb and Wood, 2019).

Nonetheless, person-focused, rather than area-focused, uses for predicting risk modeling are widespread. Current technological applications of this thinking include assigning individuals to risk groups on the basis of shared attributes (Gandy, 2020). These risk groups are often created on the basis of correlations generated algorithmically when large data sets are processed (Linder, 2019).

Such strategies come with a range of challenges and ethical gray areas, as, unlike community-based policing strategies which aim to build communities' support for the police, profiling and hotspot-oriented efforts are not always met with acceptance as they result in the accumulation of foci on poor and minority communities (Bennett Moses and Chan, 2018; National Academies of Sciences, Engineering, and Medicine, 2018; Gandy, 2020). However, the issue is complex, and defendants of proactive strategies point out that delivering police services to where the problems are is essential, and, in some countries, such as South Africa, the inability of police to enter certain neighborhoods is a contributor to inequality (Clark, 2018; Kiewit, 2019). Such efforts would ultimately need to be and be seen as legitimate and effective. There are also, naturally, sociological and political concerns about the panoptic function of these systems (Zuboff, 2015, 2019; Andrejevic, 2019).

Another application of these technologies in the crime and policing space concerns the use of algorithms in the US criminal justice system (Rizer and Watney, 2018). Evidence-based sentencing (EBS) tools, for instance, give defendants a score that represents an algorithm's prediction of recidivism based on criminal history records, employment status and history, and circumstances of the present crime (Rizer and Watney, 2018). While Rizer and Watney (2018) note that the algorithms currently used in the US pre-trial jail system are quite simple, they propose that using more advanced algorithms and ML can improve pretrial decisions and reduce the rate at which dangerous criminals are released on bail, and low-risk individuals are placed in jails which then become overcrowded and violence. The Harm Assessment Risk Tool (HART) (Oswald et al., 2018), developed by Cambridge scholars, which predicts an arrested person's risk of committing a crime if released before trial, is a good example of such a tool.

Naturally, risk-assessment algorithms must meet certain legal thresholds and be validated extensively, and they are no alternative to good judgment, but their potential to inform future decision-making is substantial, particularly—perhaps—in overwhelmed bureaucracies.

Finally, some AI exist which have been designed to recognize violence in more private spaces, including AIs which are developed to predict (and prevent) domestic and intimate partner violence (Losilla et al., 2016; Petering et al., 2018). Choo et al. (2018) developed guidelines for the establishment of a child abuse prevention system using Big Data from South Korea. The authors suggested that a child abuse eradication system could be developed for the country, using public data and big data to prevent child abuse (Choo et al., 2018). The guidelines could provide a useful template for such a project, however, whether it will result in efforts to pilot the system is unclear.

Gracia et al. (2017) conducted a 12-year (2004–2015) study using Bayesian spatiotemporal modeling and disease mapping methods to produce area-specific risk estimations for child maltreatment. Their approach could be used to improve detection of ecological variations in risk for child maltreatment, and to assess the effectiveness of the initiatives aimed at addressing risk (Gracia et al., 2017). The same authors later analyzed whether there was a common spatial distribution of child maltreatment and intimate partner violence and whether the risks of both forms of violence were influenced by the same neighborhood characteristics, and if these risks spatially overlapped (Gracia et al., 2018). Their model showed that certain neighborhood characteristics are associated with an increase in the risk of family violence, including both violence against children and against intimate partners (Gracia et al., 2018).

Other challenges are posed by the widespread recording of crime-related data is that—in the US at least—such data is now largely stored on cloud-based evidence-management systems including Evidence.com by Axon (Wood, 2017). As Gandy (2020) notes, determining who owns this data and trying to find solutions to the issues raised by the privatization of much of this technology is complicated at best.

However, Gandy (2020) also points out that statistical surveillance (unlike surveillance at the level of people, based on footage or personal sets of behavioral data) relies on powerful computing and high-level data analysis (Cheney-Lippold, 2018). Such large datasets are capable of generating a wide range of predictions about violence and crime, but limitations also exist; the data informing these predictions do not come from purpose-built, predetermined, or well-designed datasets but are largely cobbled together from a wide range of environmental sensors and other preexisting sources (Ferguson, 2017).

Now, while there are significant criticisms of all forms of predictive policing technologies (area-focused, person-centered, and statistical), their potential to assist over-stretched, low-resourced police departments and governments cannot be ignored. Neither should they be discounted because they have hitherto had certain flaws (such as drawing on poor datasets).

Fewer ethical challenges are engendered in efforts to use population-level data to make population-level predictions, than in individual-level work. Individual and system-level predictions have different pros and cons, with the former offering more specificity and more ethical challenges, and the latter less specificity, but also raising fewer issues in relation to autonomy and privacy. A possible compromise between the two models could entail using high-quality, individual-level data to develop a detailed system- or population-level model. In relation to child maltreatment, such a model could indicate—using quality cohort data, for instance—where abuse is most likely to occur and how resources should be allocated, rather than intervening in the individual case from which the prediction is generated.

Next, there are concerns about profiling. Although law enforcement in the United States, at least, already utilize social media strategies to reduce homicide rates, the use of social media data can be punitive and lead to ethical concerns regarding the impact of surveillance on communities (Patton et al., 2018). Algorithms lack the ability to accurately interpret off-line context, and there has been recognition by researchers that this could lead to the prejudicial treatment of marginalized communities. If a certain pattern of speech, for instance, at a population level is associated with criminal actions, and that speech pattern happens to be germane to a given social group, an algorithm would not necessarily be able to distinguish harmless patterned speech from the referent group, from a genuine threat. This would lead to an over-identification of potential threat among linguistic subgroup members, in this example. Some solutions to this issue have been proposed; Frey et al. (2018) worked with formerly gang-involved youth as domain experts for contextualizing Twitter data from gang-involved young people in Chicago (Frey et al., 2018). The goal of the initiative is to improve algorithms and prevent potential biases against marginalized communities being built into the AI.

Finally, experts have raised the issue of algorithmic accuracy and expectations for AI. Particularly in domains where data is lacking—for instance, in our knowledge of big expressed emotion as relates to intent to commit child abuse—an prospective actuarial formula, AI, or expert system will not be informed by a sufficient quantity of empirical research to accurately appraise risk and thus likely produce an inaccurate risk assessment. Human decision-making is not yet obsolete, and commentators raise the issue of prematurely deferring to an AI: when technology is used to determine risk, there is chance that users may discount their own knowledge of risk and over-depend on a computer-generated decision even if that decision is flawed (Reddy et al., 2001).

#### Agent-Based Modeling

An agent-based model (ABM) is a type of computational model which can be used to simulate the actions and interactions of autonomous agents (like people or companies) and in so doing assess the effects of different actions by different actors, on the system as a whole. Agent-based models (Groff et al., 2019) are increasingly used to examine population-level effects of preventive interventions with often complex causal chains such as, for example, change in closing times of alcohol outlets on conflicts in public space or effects of change in police officer allocation on violence hotspots (Groff et al., 2019). There are also many examples of such models being applied beyond the realm of policing, in public health (Maglio and Mabry, 2011), and more broadly in violence science (Epstein, 2002; Kuznar and Sedlmeyer, 2005; Lemos et al., 2013). Agent-based models, as with space-based approaches, can also be used to predict how the location of services in a city could be optimized so that at-risk individuals have the shortest and most convenient access to services.

#### Using Social Media and Other Internet Data for Violence Prevention

Twitter has 330 million active users, Facebook 2.5 billion users, and Instagram 1 billion. These individuals, on these platforms, generate millions of data points (tweets, posts, shares, and images or videos) each hour of each day. It is perhaps unsurprising, then, that researchers interested in developing machine-learning algorithms turned very early to social media for input data.

In application to the problem of violence, the use of social media data has been largely confined to natural language processing. Using these methods, social media data can provide interventionists with substantial amounts of information about marginalized communities at risk for violence (Patton et al., 2018).

The SAFE research laboratory at Columbia University, led by Desmond Patton, has developed natural language processing algorithms to identify expressed emotion (particularly focused on grief and aggression) in Twitter data from gang-involved youth. The researchers hold that such data could provide an early indication of violent intent and predict future violence (Blevins et al., 2016). Similarly, New York's Cure Violence E-Responder initiative trains individuals to identify high-risk social media content and de-escalate potentially violent conflicts online across sites in New York City^2^.

Similarly, Lee et al. (2018) developed a decision system which analyzes online language to identify cyber violence. The researchers' innovation is novel in that it can detect cases where abuse language (abusive language, slang, and profanity) has been obfuscated (the problem words hidden or other terms used in their place). The integrated decision system which Lee et al. (2018) developed showed a precision of 94.08% in malicious word detection for news article comments, a precision of 89.97% in malicious word detection for online community comments, and a precision of 90.65% in malicious word detection for Twitter tweets.

Other work, by Zarnoufi and Abik (2019) used a set of ensemble learning algorithms with engineered features related to the vocabulary used frequently in each Big Five personality trait (Agreeableness, Conscientiousness, Extraversion, Neuroticism and Openness), to identify individuals with harmful intention online. The findings show a significant association between the individuals' personality state and their perpetration of cyber violence (Zarnoufi and Abik, 2019).

It is worth noting that, in relation to many of the above examples, the use of AI expert decision-making systems has raised concerns regarding privacy, profiling, and the creation of expectations that exceed what expert systems can reasonably do, with accuracy (Reddy et al., 2001). Regarding privacy, there are widespread and well-founded issues in the use of personal, albeit public domain, data to make predictions about human behavior. If an individual shows intent to conduct and action, for instance, and then an algorithm flags them for intervention, but they had no desire for intervention nor real intent to engage in the flagged action, they could be marked or stigmatized by the intervention, without due cause. More generally, it is widely debated whether monitoring individual data, even for the greater good, is ethical, especially when those being monitored are unaware that they are being surveilled.

### mHealth Technology in Prevention, Early Detection, and Early Response to Violence, Including Violence Against Children

mHealth in violence prevention has, in the main, taken three forms, all of which entail varying degrees of intervention; one, simply hosting information (such as websites which have repositories of safety information or support systems for violence-affected persons); two, delivering novel interventions to young people at risk of perpetrating or being victims of violent behavior; and three, augmenting existing preventative interventions targeted at reducing violence against children.

#### Providing and Hosting Information

Simple informational websites or anonymous support systems for victims represent the digitization of channels which have long existed, but which may not have been accessible (for reasons of stigma, resources or logistics) to people who have experienced violence. While this may not constitute mHealth as it is commonly thought of (apps), these web-based services (traditionally defined as eHealth) do represent a novel and important digital contribution to violence prevention, and responses to violence.

#### Delivery of Intervention Programs

Given the widespread penetration of smartphones and personal computers globally, a large number of mHealth applications have been developed in the past decade (and some before) to deliver interventional content directly to end users, through their phone or PC.

Social skills training (SST) programs—an evidence-based means of improving children's social skills and behavior—has been digitized in the intervention Zoo U. This game-based SST program facilitates the development of prosocial skills among children in an attempt to prevent violence (Craig et al., 2016). Similarly, KiVa (http://www.kivaprogram.net/) is a program that uses a lot of games, videos, and web-hosted information systems for teachers to prevent bullying.

There are also examples of mHealth parenting and parent-training interventions (Breitenstein et al., 2017), which similarly intervenes directly through a technological platform, to change behavior. Among these parent interventions relevant to violence prevention are those which seek to address adult mental health problems. Ginger (https://www.ginger.io/) provides access to evidence-based behavioral health coaching, video therapy, video psychiatry, and self-guided content for mental health conditions. Ginger's algorithms analyze the words which users use in relation to its “knowledge” of 2 billion behavioral data points, 45 million chat messages, and 2 million clinical assessments, to provide a recommendation for treatment to users (Hunt, 2019; Marr, 2019).

The WoeBot (https://woebot.io/), for instance, is an AI-drive app, which provides “quick conversations to feel better,” for individuals feeling isolated or sad. Stanford psychologists built basic cognitive behavioral therapy (CBT) algorithms into the automated “bot” system, which is downloadable as an app. The system then provides brief, evidence-based chat responses to individuals who type their thoughts, feelings, and other content into the app's chat. Randomized controlled trial evaluation of the app has shown a reduction in depressive symptoms over a short period, amongst adult users (Fitzpatrick et al., 2017). Quartet (https://my.quartethealth.com/how-it-works/) allows online users to take a short “wellness assessment” to flag possible mental health conditions and can refer users to a provider or a computerized cognitive behavioral therapy program based on the results.

On the prevention front, Bark (https://www.bark.us/) and FamiSafe (https://famisafe.wondershare.com/) allow parents to monitor children's phones, accessing major messaging and social media platforms to look for signs of cyber bullying, depression, suicidal thoughts, and sexting on a child's phone (Marr, 2019). Similarly, Ferreira et al. (2020) conducted a review of mHealth apps for reporting violence in schools, using a benchmarking tool to produce an integrative review of the software. However, the process showed that most of the applications lacked a comprehensive array of evidence-based violence prevention and response features.

#### Augmenting the Effects of Existing Interventions

Thirdly, concerning the augmentation of existing preventative interventions targeted at reducing violence, a recent systematic review (Cronin et al., 2017) of social and behavior change interventions utilizing technology to address violence against children yielded 18 papers on the topic. The authors noted that the majority of intervention typically combined technological intervention or mHealth components, with other, traditional communication channels in order to reach children, parents, teachers, or other child service providers. Examples of such interventions included a mobile phone-enhanced intervention for families at risk for child neglect (Bigelow et al., 2008), and a mobile phone enhancement of a parenting intervention to address child maltreatment (Jabaley et al., 2011). The authors lamented the strength of available evidence and pointed to the need for rigorous evaluations to assess the utility and value of mHealth components in violence prevention and intervention.

Anderson et al. (2019) similarly systematically reviewed the literature on mHealth interventions, including web- or mobile-based delivery methods for primary, secondary, and tertiary intimate partner violence victimization prevention. The review yielded 31 primary studies, the majority of which concerned computer-based screening, followed by decision aids. In many of the studies, the interventions were found to be both feasible and acceptable. Yet, the authors cautioned that there was limited evidence around whether mHealth interventions better addressed population needs compared to conventional interventions. Nonetheless, they noted that a major strength of mHealth IPV prevention programming lay in its potential to tailor interventions to individual victim needs without the requirement of extensive human resources.

One study has evaluated healthcare workers' and women's perceptions and experiences of using the Domestic Violence Enhanced Home Visitation Program (DOVE), and mHealth technology as compared to a home visitor-administered, paper-based method during perinatal home visiting (Bacchus et al., 2016). The authors noted that in respect of such a sensitive topic as IPV, the importance of the patient–provider relationship in promoting behavior change needed to be recognized by interventionists, and—as such—mHealth approaches should be used to complement and enhance the therapeutic relationship, rather than replace it (Bacchus et al., 2016).

## Discussion

### The INSPIRE Framework

It is currently unclear how AI-based and mHealth-based strategies could help to inform a comprehensive violence prevention strategy. Mostly suggestions have been limited to one particular approach, i.e., either pattern recognition or dissemination of parenting etc.

In the next section, we hence explore how the various strategies described in the previous section can help to support an integrated violence prevention strategy, mainly in LMICs. Table 1 briefly summarizes the contributions of these technologies to violence prevention priorities. For this purpose, we focus on INSPIRE, the World Health Organization's framework of strategies to prevention violence against children (World Health Organization, 2016). INSPIRE is a widely used integrated strategy based on public health principles, as it seeks to address violence through a multisectoral response and multipronged set of actions. The strategies include implementation and enforcement of laws; norms and values; safe environments; parent and caregiver support; income and economic strengthening; response and support services; and education and life skills. We examine, for each of the seven strategies, how and whether AI and mHealth could work to address the priorities highlighted by the framework.

**Table 1:** Mapping the possible contributions of AI, machine learning, Big Data, and mHealth.

**What**	**What can it contribute?**
**AI/Machine learning and big data**	
Training of Public Health Practitioners	Interactive AI-based learning tools can help to train healthcare providers in professional help for victims of violence and abuse.
Cost-effective provision of more intensive indicated prevention	Where big data are available machine learning can be used to identify subgroups for whom more intensive preventive interventions are most likely to be helpful.
Modeling of population-wide intervention effects	Agent-based models can help to better understand the effects of interventions at the population level, especially if interventions intervene in several systems simultaneously.
Clearance of violent crimes	Almost all crimes in HICs leave an electronic footprint, and this will be increasingly the case in LMICs.
Recognition and Prevention of Violence in Cyberspace	Can protect vulnerable children from exploitation online
Place-based policing	Can inform the allocation of resources to bridge inequities in allocation of prevention and response resources
Risk prediction for reoffending	Machine learning could help to optimize assessments for re-offending risk.
**mHealth**	
mHealth-based interventions for children and adolescents with behavior problems	Lack of child psychiatrists and psychologists in many LMICs. mHealth-based platforms can provide basic universal coverage.
mHealth-based interventions for victims of violence, abuse, and bullying including emergency lines and information systems, possibly developing into expert systems	Lack of child psychiatrists and psychologists in many LMICs. mHealth-based platforms can provide basic universal coverage. Expert systems could aid professional capacity development of frontline workers in violence response, given dearth of professional staff.
mHealth interventions for parents and carers	Parenting and childcare advice at different levels of sophistication.

Corresponding to each of the INSPIRE pillars are objectives. These objectives should guide priority action in efforts to support the core element of the pillar (see Table 2).

**Table 2:** The INSPIRE objectives.

**INSPIRE pillar**	**Objective**
Implementation and enforcement of laws	Ensure the implementation and enforcement of laws that prohibit and prevent violence against children, reduce excessive alcohol use, and limit youth access to firearms and other weapons.
Norms and values	Strengthen norms and values that support non-violent, respectful, nurturing, positive, and gender-equitable relationships for all children and adolescents.
Safe environments	Create and sustain safe physical and social environments where children and youth gather and spend time.
Parent and caregiver support	Reduce harsh parenting practices and create positive parent-child relationships.
Income and economic strengthening	Improve family economic security and stability and reduce child maltreatment and intimate partner violence.
Response and support services	Improve access to good-quality health, social welfare, and justice support services—including reporting violence—for all children who need them, to reduce the long-term impact of violence.
Education and life skills	Increase children's access to more effective, gender-equitable education and social-emotional learning and life skills training, and ensure that school environments are safe and enabling.

### Mapping Solutions to the Gaps: Innovations in Violence Prevention

The literature on AI, big data, and mHealth applications for violence prevention and response, clearly aligned to one or more INSPIRE strategies. Table 2 lists the INSPIRE objectives, and Table 3 lays out a visual representation of the possible solutions offered by AI, big data, and mHealth yielded by our review, mapped onto the INSPIRE pillars.

**Table 3:** Mapping violence prevention priorities and possible AI, mHealth, and machine-learning solutions onto the INSPIRE objectives.

**INSPIRE pillar**	**INSPIRE intervention foci**	**Example of AI-, machine learning, and Big Data-driven solutions**	**mHealth interventions**
Implementation and enforcement of laws	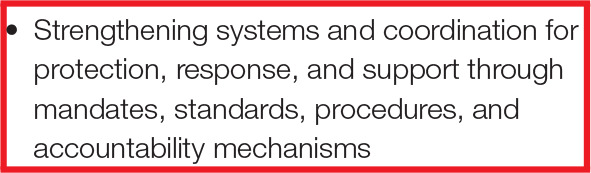 • Establishing frameworks for identification, referral, investigation, treatment, and follow-up for children who experience violence • Establishing pathways to fair, transparent, and child-friendly justice for all children	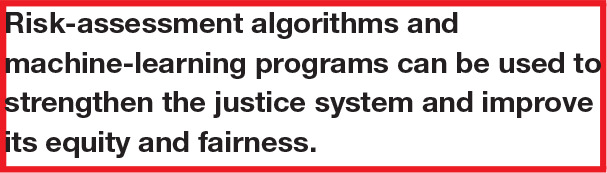	
Norms and values	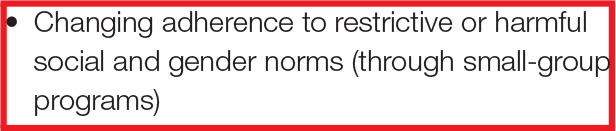 • Community and bystander mobilization		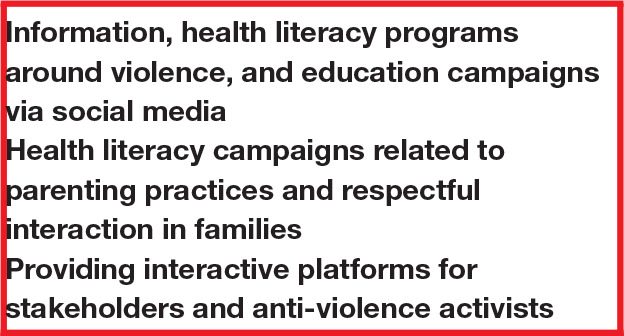
Safe Environments	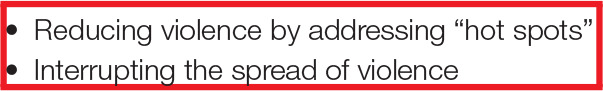 • Improving the built environment	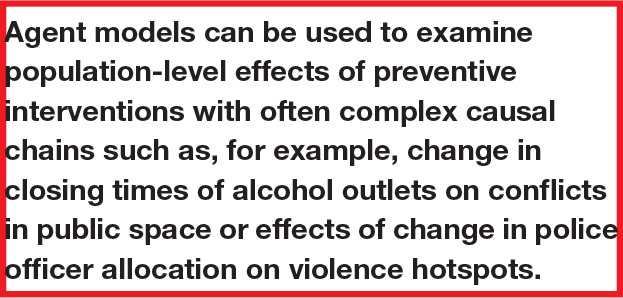 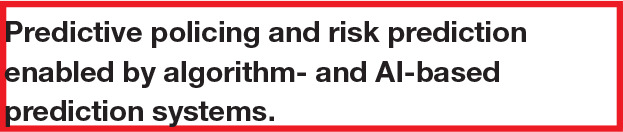 Urban planning approaches to create safe environments for children supported by AI, GIS, etc.	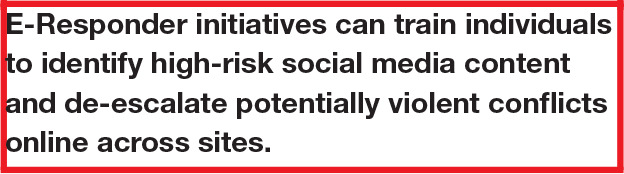
Parent and caregiver support		Some AIs have been developed to predict (and prevent) domestic and intimate partner violence. There are guidelines (rather than an intervention) for the establishment of a child abuse prevention system using Big Data. 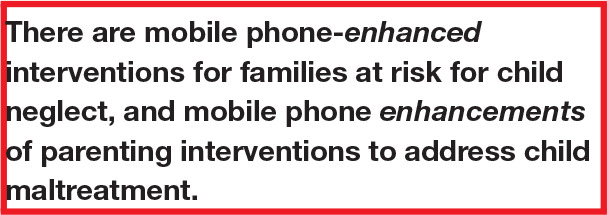	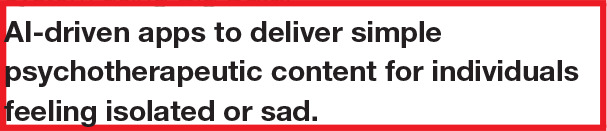
Income and economic strengthening	• Income and economic strengthening (IES) • IES efforts to target women, focusing on reducing poverty, improving child health and nutrition, supporting education, or empowering women generally	Online training and qualification programs for vulnerable groups? Information	
Response and support services	• Have a system for helping children 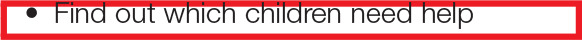 • Help children immediately and in the longer-term (including counseling and therapeutic approaches) • Protect children in conflict with the law	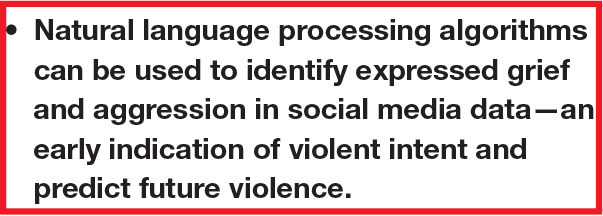 • Risk prediction and tailoring of interventions for victims of IPV and VAC, child maltreatment • Data sharing across services for better interventions (see the Cardiff Model of collaboration between police and public health → Jonathan Shepherd	
Education and life skills	• Support school participation • Create safe and supportive school environments 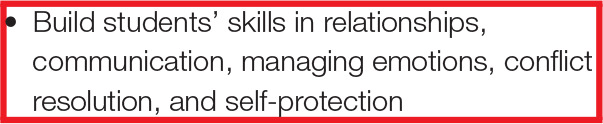		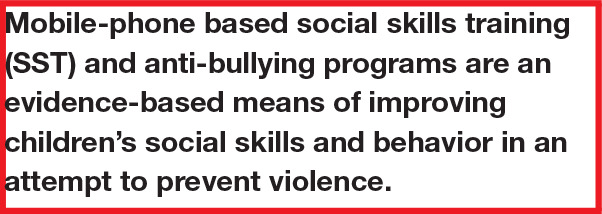

*Examples where possible solutions are apparent are encircled in red*.

## Directions for Policy and Programming for AI, Big Data, and mHealth in Violence Prevention Work in LMIC

Certain clear directions for applications of these technologies in low- and middle-income countries emerge out of the foregoing discussion, and these are summarized below.

### AI, Big Data, and mHealth: Closing or Widening Inequalities

There is substantial potential for AI (and associated machine learning and big data), and mHealth, approaches to be utilized to prevent and address violence at a large scale. This potential is particularly marked in low- and middle-income countries (LMIC). However, such work should be approached with caution.

Indeed, the potential of mHealth, as has been widely proposed (Chang et al., 2011; Chib et al., 2015; Morgan et al., 2017), could lie in its capacity to reach underserved populations (although current analyses have cast some doubt on this, see Jennings and Gagliardi, 2013; Morgan et al., 2017). Resource constraints are endemic in many low- and middle-income countries and hinder public health efforts. A substantial number of mHealth interventions have thus been implemented in LMIC public health in the past decade, premised on the assumption that such technologies can bridge the resources divide (Chang et al., 2011; Chib et al., 2015; Morgan et al., 2017). However, as commentators have noted, mHealth interventions alone are unlikely enough to achieve real behavior change, in contexts where economic, social, political, and infrastructural barriers to service access and behavior change are unlikely to be addressed by messaging alone (Morgan et al., 2017). Where mHealth could provide real solutions, we would offer, would be in cases where the mHealth product itself increases resource access (either by being a resource or intervention itself, such as the WoeBot) or connecting individuals to services which they would not otherwise be able to reach.

The role of AI and big data in relation to inequalities is less apparent but nonetheless important. As more and more decision-making involves AI, it will be imperative that there are ongoing efforts to minimize harm and maximize benefits. As a series of now-canceled technologies have shown, AI has a tendency to absorb existing biases: Amazon, for instance, had to recall an AI which was used in recruiting when it began to show a tendency to preferentially hire men (Dastin, 2018; Posner, 2018). Similarly, the US Attorney General (in 2014) noted that the risk assessment algorithms used in the justice system may “exacerbate unwarranted and unjust disparities that are already far too common in our criminal justice system and in our society” as they assign higher risk scores to people of color (Angwin et al., 2016; Posner, 2018).

There is, as has been noted by commentators (Angwin et al., 2016; Posner, 2018), an urgent need to ensure that bias is not built into so-called “expert decision systems.” This will require, at least, very careful designing of AI programming, and ongoing monitoring of their functions for evidence of bias (Posner, 2018).

However, more broadly than simply ensuring good design and monitoring, the fourth industrial revolution must be an inclusive one. Broadening access and inclusion for AI may go some way to mitigate the risks of inequality in their application, but should contribute to the optimization of the technologies themselves (Posner, 2018); if quality and representativeness of data in means accuracy and specificity of predictions out, inclusion is the only way for AI to reach their potential.

There must be concerted, meaningful efforts to develop participatory and user-led work in the AI space, to ensure that the privacy and profiling concerns outlined above are addressed explicitly going forward. Hopefully such efforts would also address issues around cultural and contextual fit of whichever solutions are implemented.

Finally, although their potential to lessen some inequalities in health and social development, much of the evidence for innovations discussed in this paper is not based on findings from scaled up or widely implemented iterations of these interventions. Evidence of effectiveness in real-world settings will be imperative to assess the applicability and likely utility of these solutions to LMIC.

### Bolstering Input Data to Improve Utility

Given that AI is only as good as the data informing it, there is a need to develop common, reliable, and valid population and individual/family-level data on predictors of violence. These indicators could be integrated into routine health or other information systems and become the basis of Al algorithms for violence prevention and response systems. Further, data on individual help-seeking behavior, risk factors for child maltreatment, and other information which could help us to identify the parameters required to understand what happens to cause and, in response to violence, are needed.

As noted in a recent report by the Medical Academy of Royal Colleges (2019), good-quality AI depends on good-quality data, and “with a few notable exceptions, the quality of patient level data is notoriously patchy (in the NHS).” This comment is based on concerns in a very high-income country: the United Kingdom. In many LMIC, national health information systems (NHIMS) are weak and limited. However, given the international drive toward universal health coverage (UHC) (Mukherjee et al., 2019),^3^ many LMIC are investing in efforts to strengthen NHIMS and routine data collection. As noted by WHO, “comprehensive, timely, and reliable health and health-related statistics are fundamental for assessing the state of a population's health and how it is changing” (WHO RGHS, 2020), and this is even more so the case when such data is going to be used to inform planning, decisions, actions and resource-allocation at scale.

At present, there are well-documented limitations in national information systems (Bosch-Capblanch et al., 2009; Upadhaya et al., 2016; MEASURE Evaluation, 2018), and a general lack of resources for big data-driven innovations in LMIC. As such, developing AI and other technological infrastructure will require substantial investment, particularly in LMIC, in existing data collection systems.

### Applications Beyond Intervention

AI, Big Data, and mHealth strategies can be leveraged not only as interventions but also as evaluative tools to assess real-world programs, for instance using agent-based modeling when innovative general prevention strategies are introduced into existing systems. If we want to inform policymakers about the multiple effects of system-wide change, we could benefit a lot from agent-based modeling.

For instance, it is fairly well-established at the program level, including in LMIC, that intervention implementers and healthcare workers can effectively use mobile phones for data collection and surveillance (Barrington et al., 2010; Hoffman et al., 2010; Kaewkungwal et al., 2010; Andreatta et al., 2011; Muthiah et al., 2011; MacLeod et al., 2012; Blank et al., 2013; Chaiyachati et al., 2013; Githinji et al., 2013; Agarwal et al., 2015; Soltanipoor et al., 2019). Mobile-based data collection improves promptness of data collection, reduces error rates, and improves data completeness (Agarwal et al., 2015).

### A Focus on Strengthening Existing Systems

Globally, there is a shortage of 7.2 million healthcare workers. This need is particularly pronounced in LMIC. Various mHealth applications have been developed to support the paraprofessional health workers who have been employed in resource-scarce settings to bridge this service gap (Agarwal et al., 2015). However, as Labrique et al. (2013) note, rather than being seen as “silver bullet” stand-alone solutions, mHealth strategies should be fit for integration into existing health system functions. Rather than trying to make a mobile phone application meet the violence prevention needs of under-resourced communities (unlikely), they could better be used to expand and strengthen the existing goals, platforms and practices of violence prevention infrastructure.

Agarwal et al. (2015)—in their review of the literature on mHealth in healthcare systems—noted that the use of mHealth strategies by frontline healthcare workers *might* offer some promising approaches to improving healthcare delivery but that the evidence on the effectiveness of such strategies on healthcare outcomes was insufficient at the time of writing. Use of mHealth strategies could potentially circumvent several of the structural and systemic barriers faced by frontline workers in violence prevention and response—in delivering services. Evidence from healthcare shows that the use of mobile phones for service delivery is feasible for frontline workers irrespective of their education or prior training. However, a majority of the studies in this area are in the field of health, only pilot activities, and provide minimal information about the effectiveness of the use of mHealth tools on the quality and efficiency of services. However, evidence from low-resource settings, presented in a review by White et al. (2016), suggests that mHealth does have utility in LMIC and noted the potential for widespread health system improvements using technology.

Finally, Lin et al. (2019), in a recent article, describe how artificial intelligence could transform primary care. The researchers highlight a number of strengths, or which five are pertinent to the present discussion on violence in LMIC settings.

Firstly, they note that AI could fundamentally shift in how resources are allocated. As noted above, targeting is important in settings where resources for intervention are scarce. It is important that those who are most vulnerable have access to services. With better risk prediction in primary care settings—and even at the population level—resources could potentially be better allocated to priority patients or participants. For instance, some of the promise of risk prediction and violence prevention has to do with caregiving capacity and child maltreatment prevention. If caregivers are found to be at risk of poor parenting practices, they can be targeted for targeted prevention efforts. Offline, efforts to identify children at risk are often informed by ACE—a measure of childhood adversities and trauma which is an estimate for the likely need of more intensive preventive intervention (Larkin and Park, 2012; World Health Organization, 2018). It is plausible to imagine that the ACE domains (prior to the age of 18: physical abuse; emotional abuse; sexual abuse; domestic violence; growing up with a substance abusing household member; living with a mentally ill/suicidal household member; experiencing the incarceration of a household member; loss of a parent; emotional neglect; or physical neglect) (Larkin and Park, 2012; World Health Organization, 2018) could be used to populate algorithms to identify children at risk at a much larger scale.

Next, also of relevance to violence in LMIC, Lin et al. (2019) propose that population health management can be enabled by AI. AI, they argue, has the potential to identify and close care gaps. In the first instance, we have discussed, above, how health inequalities can be mapped spatially, and how this can provide clear guidance for equity-focused resource allocation. In relation to violence, Big Data could play a similar role, highlighting where the violence to resources to respond to it ratio is inequitable.

Clinical decision-making is another area where the potential contributions of AI at the frontlines could be made. Were effective AI to be added to workflows for police, child protection personnel, social workers, and other interventionists—particularly where such personnel are scarce in LMIC—much-needed decision support could be widely available.

Finally, direct-to-user applications, such as “advice and triage” and digital coaching, are highlighted by Lin et al. (2019). Both of these have—in the field of health—been delivered through mHealth applications. As described by Morgan et al. (2017), however, the limitations of such technologies in LMIC need to be addressed if mHealth is to provide sustainable solutions. In essence, as promising as tech-delivered interventions may be, if they are not supported by actions to address the social determinants of health and violence, it is unlikely that they will be effective in the long term. Further, if such interventions are scale in LMIC in the absence of proper infrastructure, their impact will likely be limited.

Engelhard et al. (2018) report on South Africa's national-level helpdesk which was established in 2014 as a social accountability mechanism for improving governance, allowing recipients of public sector services to send complaints, compliments, and questions directly to a team of National Department of Health staff members via text message. Focusing on messages related to the mistreatment of women, the authors noted that current response to a specific, high-priority topic—the mistreatment of women—is no better than its response to the average incoming message (Engelhard et al., 2018). Given the high volume of messages, the authors note, this is to be expected. They show how an automated triage system which sorts incoming messages by priority could improve the timeliness and appropriateness of helpdesk responses.

In a commentary for the Lancet Public Health, Jewkes and Dartnall (2019) discussed the proliferation of web-based interventions for violence against women, critiquing the lack of an associated evidence base to accompany their roll-out. The authors called out the “inadequate investment in formative research” prior to the development of these technologies and noted that the lack of evaluation is particularly notable in LMIC. In a context where funding for prevention of and responses to violence against women is scare, they cautioned, a critical appraisal of the opportunity costs of scaling innovations without evidence of effect, is urgently required.

This review shows that there continues to be a lack of evidence for effectiveness at scale and an ongoing failure to quantify the systems and inputs requirements needed for these technologies to be impactful in LMIC. However, this review also undoubtedly showcases the potential of AI-, Big Data-, and mHealth-driven interventions to bridge gaps in services in LMIC, improve violence prevention, and bolster individual, community, and national responses to violence, including violence against children. The case for investment for further research, at least, seems clear. However, this research must inform and precede, scale, if the problem of violence, including violence against children, is to be sustainably addressed.

## Author Contributions

ME and XH developed the idea for the paper, with input from MT and SSi. XH, MT, and ME conducted the scoping of the literature and developed the paper framework. MM, SdT, and SSk worked on manuscript drafts developed by XH. ME and MT provided ongoing input and support to XH in the refinement of the piece. All authors contributed to the article and approved the submitted version.

## Conflict of Interest

The authors declare that the research was conducted in the absence of any commercial or financial relationships that could be construed as a potential conflict of interest.

## Footnotes

^1^Natural language processing is the application of computational techniques to the analysis and synthesis of natural language and speech. Computer programs analyze large amounts of language (from books or social media, for instance) to arrive at a specific outcome, for instance a prediction about the types of language used in certain situations, or the most commonly co-occurring words.

^2^Researching Inequity in Society Ecologically (R.I.S.E.) Team. “E-Responder: a brief about preventing real world violence using digital intervention.”

^3^World Health Organization. “Universal Health Coverage,” from https://www.who.int/health-topics/universal-health-coverage#tab=tab_1.
